# Effects of unburned tobacco smoke on inflammatory and oxidative mediators in the rat prefrontal cortex

**DOI:** 10.3389/fphar.2024.1328917

**Published:** 2024-01-25

**Authors:** Fabio Vivarelli, Camilla Morosini, Laura Rullo, Loredana Maria Losapio, Antonio Lacorte, Stefano Sangiorgi, Severino Ghini, Ivan Fagiolino, Paola Franchi, Marco Lucarini, Sanzio Candeletti, Donatella Canistro, Patrizia Romualdi, Moreno Paolini

**Affiliations:** ^1^ Department of Pharmacy and Biotechnology, Alma Mater Studiorum–University of Bologna, Bologna, Italy; ^2^ Gruppo CSA–S.p.A Via al Torrente 22, Rimini, Italy; ^3^ Department of Chemistry “G. Ciamician”, Alma Mater Studiorum–University of Bologna, Bologna, Italy

**Keywords:** e-cigarette, heat-not-burn, oxidative stress, inflammation, prefrontal cortex, peroxisome proliferator-activated receptors, KDMs

## Abstract

Although the Food and Drug Administration has authorized the marketing of “heat-not-burn” (HnB) electronic cigarettes as a modified risk tobacco product (MRTP), toxicological effects of HnB smoke exposure on the brain are still unexplored. Here, paramagnetic resonance of the prefrontal cortex (PFC) of HnB-exposed rats shows a dramatic increase in reactive radical species (RRS) yield coupled with an inflammatory response mediated by NF-κB-target genes including TNF-α, IL-1β, and IL-6 and the downregulation of peroxisome proliferator-activated receptor (PPAR) alpha and gamma expression. The PFC shows higher levels of 8-hydroxyguanosine, a marker of DNA oxidative damage, along with the activation of antioxidant machinery and DNA repair systems, including xeroderma pigmentosum group C (XPC) protein complex and 8-oxoguanine DNA glycosylase 1. HnB also induces the expression of drug-metabolizing enzymes such as CYP1A1, CYP2A6, CYP2B6, and CYP2E, particularly involved in the biotransformation of nicotine and several carcinogenic agents such as aldehydes and polycyclic aromatic hydrocarbons here recorded in the HnB stick smoke. Taken together, these effects, from disruption of redox homeostasis, inflammation, PPAR manipulation along with enhanced bioactivation of neurotoxicants, and upregulation of cMYC protooncogene to impairment of primary cellular defense mechanisms, suggest a possible increased risk of brain cancer. Although the HnB device reduces the emission of tobacco toxicants, our findings indicate that its consumption may carry a risk of potential adverse health effects, especially in non-smokers so far. Further studies are needed to fully understand the long-term effects of these devices.

## 1 Introduction

Tobacco addiction represents a widespread and severe public health problem that results in high morbidity and mortality (Soares and Picciotto, 2023). Indeed, smoking is the primary cause of chronic obstructive airway disease, and it is accounts for more than 90% of lung cancer cases along with the marked impact on the incidence of cardiovascular diseases, including coronary heart disease (WHO, 2023a). Electronic cigarettes (e-cigarettes; e-cigs) were conceived as a safer alternative to tobacco products and have also been proposed as a possible approach to smoking cessation even though they have never been approved for this latter purpose by the Food and Drug Administration (FDA) (Wang et al., 2021). In particular, the last generation of these devices, called “heat-not-burn” (HnB), has achieved great popularity in recent years, especially among young people (Vivarelli et al., 2022). Equipped with a technology that heats tobacco without combustion, these devices are marketed as harmless and high-tech nicotine-delivery tools, targeting current smokers, ex-smokers, and young people who have never smoked. Although the HnB mainstream has shown a lower concentration of toxic and carcinogenic components than conventional cigarettes, such as aldehydes and carcinogenic polycyclic aromatic hydrocarbons (PAHs) (Vivarelli et al., 2021), and these electronic tobacco devices are currently marketed as “modified tobacco risk products,” evidence from cellular and animal models underscore lung tissue damage, genotoxicity, and carcinogenic risk (Vivarelli et al., 2021; Sawa et al., 2022). Literature suggests that smoking habit shows deleterious neurological effects via oxidative stress, triggering inflammation via a cytokine-mediated immune response with the release of pro-inflammatory mediators such as TNF-α, IL-1, and IL-6 that may exert neurotoxic effects (Chitnis and Weiner, 2017) accelerating brain aging. In this frame, the crucial role of peroxisome proliferator-activated receptors (PPARs) has been established (Domi et al., 2019; Sivandzade and Cucullo, 2019). Indeed, there is consistent evidence that these nuclear receptors are involved in the modulation of transcription factors such as nuclear factor erythroid 2-related factor (NRF2) and nuclear factor kappa-light-chain-enhancer of activated B cells (NF-κB), known to be involved in antioxidant as well as inflammatory responses (Caputi et al., 2019; Korbecki et al., 2019). Interestingly, in recent years, it has been shown that the epigenetic enzymes involved in histone lysine demethylation (KDMs) could promote the inflammatory process through the regulation of some pro-inflammatory mediator’s gene expression such as TNF-α, IL-6, and IL-1β (Li et al., 2017; Higashijima et al., 2020; Rullo et al., 2021). Furthermore, epigenetic modifications regulated by KDMs are also reported to affect the gene expression and activity of PPARs (Rullo et al., 2021), thus supporting the role of these epigenetic mechanisms in smoke-induced inflammation. Although the implication of oxidative and inflammatory phenomena in the toxicity of conventional cigarette smoke is well known, the toxicological effects associated with HnB products on the central nervous system (CNS) are still unknown (WHO, 2023b). The prefrontal cortex (PFC) is one of the brain regions mostly affected by traditional tobacco smoking (Karama et al., 2015) and plays a crucial role in the reward circuitry activated by drugs of abuse, including nicotine (Goldstein and Volkow, 2011). On this base and taking into account the role of oxidative and inflammatory process in substance use disorder development (Zhang et al., 2018; McGrath and Briand, 2019; Johnstone et al., 2021; Guo et al., 2023), the present study aimed to investigate the neurotoxic effects induced by the exposure to the HnB tobacco stick mainstream and likely associated with tobacco dependence in the rat PFC. In particular, the generation of reactive radical species (RRS) and the perturbation of antioxidant and DNA repair enzymatic machinery were assessed. Moreover, the connection between oxidative stress, inflammation, and DNA damage (Kawanishi et al., 2017) along with the impact of HnB exposure on the bioactivating cytochrome P450 (CYP) superfamily of isoforms, cell cycle progression, and transformation factors such as c-MYC was studied.

## 2 Materials and methods

### 2.1 Heat-not-burn electronic cigarette

The heat-not-burn THS 2.2 model produced by PMI was used in the present study to deliver tobacco aerosol. The device and the tobacco sticks (HEETS Bronze) used in this study are commercially available.

### 2.2 Chemical characterization of HnB tobacco stick smoke

Chemical analysis on HnB mainstream smoke was performed as previously reported (Vivarelli et al., 2021). The compounds were analyzed using GC/MS applying the following procedures: powders inhalable fraction M.U. 1998-13; nitrogen oxides NIOSH 6014 1994; nicotine 2551 1998; aldehydes EPA8315A 1996; phenols and cresols NIOSH 2546 1994; volatile organic compounds (VOCs) UNI EN ISO 16017-1: 2002; BTEX UNI EN ISO 16017-1: 2002; metals UNI EN 14902:2005/EC1:2008 + UNI EN ISO 17294-2:2016; and polycyclic aromatic hydrocarbons DM 05/05/2015 GU n128 05/06/2016.

### 2.3 Preliminary conditions and chamber assessment

The puff profile and total tobacco stick number were set in order to ensure appropriate O_2_/CO_2_ and O_2_/N_2_ ratios with a decrease in the oxygen level (less than 5%) and a slightly higher relative CO_2_ concentration, as previously described (Canistro et al., 2017; Vivarelli et al., 2021). Air was sampled by the use of a Hamilton airtight syringe (30 mL), which was immediately transferred into a 5-mL capped vial and injected onto a GC/MS system (QP-2010 Plus, Shimadzu, Japan) equipped with an Rtx-Wax column (30 m, 0.25 mm i. d., 0.25 μm film thickness, Restek, United States), interfaced with a computerized system for data acquisition (software GC–MS Solution V. 2.5, Shimadzu, Japan).

### 2.4 Animal model

Animal experiments were set in accordance with EU Directive (2010/63/EU) guidelines, and the protocol was approved by the Committee on the Ethics of Animal Experiments of the University of Bologna and by the Italian Ministry of Health (permit number 360/216-PR; 2683215). Male Sprague–Dawley rats (Envigo RMS S.r.l., San Pietro al Natisone, Udine, Italy), aged 7 weeks, were housed under standard conditions (12 h light–dark cycle, 22°C, and 60% humidity). After 2 weeks of acclimatization, animals were randomly assigned to the control (*n* = 6) or exposed (*n* = 6) group. The exposure chamber consisted of a propylene chamber (38 × 26.5 × 19 cm) with a capacity of 19 L. The pump (0.18 kW; 1.4/1.6 A; 230 V; 50/60 Hz) was connected on the one side of the box, while the mainstream was puffed on the opposite side, generating the airflow into the chamber as described in previous studies (Canistro et al., 2017; Cardenia et al., 2018; Cirillo et al., 2019; Vivarelli et al., 2019; Vivarelli et al., 2021). Two animals were placed in the chamber, and they were subjected to a total-body exposure. The puff profile (5 s on, 15 s off, and 5 s on) with an airflow of 4 L/min was set in accordance with previous studies (Nabavizadeh et al., 2018; Vivarelli et al., 2021). The exposure lasted for 20 min. Animals were submitted to mainstream smoke from eight tobacco sticks/day/chamber, never exceeding the 3 h/day of exposure. The concentration of nicotine in the chamber was significantly lower than the LC_50_ for vaporized nicotine in the rat model (2.3 mg/L) (Shao et al., 2013). In accordance with the Animal Welfare Committee, the treatment was scheduled for 5 consecutive days/week followed by 2 rest days for 4 weeks. Experiments involving animals have been reported according to the ARRIVE guidelines. All efforts were made to minimize animal suffering and to reduce the number of animals used. Controls were not exposed to any treatment; however, the animals spent the same time in the exposure chamber as the treated animals.

### 2.5 Tissue collection

At the end of the experimental protocol, animals were sacrificed by decapitation. For each rat, the PFC was collected, as previously reported (Rullo et al., 2023) and according to the Rat Brain Atlas (Paxinos and Watson, 2013). Tissues were immediately stored at −80°C for further *ex vivo* analysis.

### 2.6 Analysis of reactive oxygen, nitrogen, and carbon species in the PFC by electronic paramagnetic resonance (EPR) spectroscopy

Biopsies were treated with 0.5 mL of standard physiological solution containing the hydroxylamine “spin probe” (bis(1-hydroxy-2,2,6,6-tetramethyl-4-piperidinyl) decandioate dihydrochloride) (1 mM), synthetized as previously reported (Fabbri et al., 2015) and deferoxamine (1 mM) as the metal chelating agent. The tubes were incubated at 37 °C for 5 min and then snap-frozen in liquid nitrogen and stored at −80°C until electronic paramagnetic resonance (EPR) analyses. The nitroxide spectra generated by the reaction of the probe with the radicals produced in the tissues were recorded using the following parameters: modulation amplitude = 1.0 G; conversion time = 163.84 ms; modulation frequency 100 kHz; and microwave power = 6.4 mW. The intensity of the first spectral line of the nitroxide (aN = 16.90 G and g = 2.0056) was considered a nitroxide amount in each examined. The calibration of the spectrometer response was done by using a known solution of TEMPO-choline in water and an ER 4119HS Bruker Marker Accessory as the internal standard (Fabbri et al., 2015).

### 2.7 8-Hydro-2-deoxyguanosine (8-OHdG) ELISA assay

The test was performed following the manufacturer’s instructions (DNA/RNA oxidative damage ELISA Kit by Cayman Chemicals, Ann Harbor, MI, United States). The DNA was extracted from tissue biopsies by the use of AllPrep DNA/RNA Kit (QIAGEN, Venlo, Netherlands; see also Section 2.8), following the datasheet recommendations. Nucleosides were obtained using the DNA Degradase Plus Kit purchased from Zymo Research Irvine, CA, United States.

### 2.8 RNA extraction and gene expression analysis by real-time qPCR

Total RNA and DNA were extracted simultaneously using the AllPrep DNA/RNA Kit (QIAGEN, Venlo, Netherlands), according to the manufacturer’s instructions. RNA integrity was checked by 1% agarose gel electrophoresis, and concentrations were measured using a NanoDrop 1000 system spectrophotometer (Thermo Fisher Scientific, Waltham, MS, United States), as previously described (Caputi et al., 2021). Each sample was subjected to conversion to cDNA using the GeneAmp RNA PCR Kit (Life Technologies Italia, Monza, Italy), according to the manufacturer’s protocol. Relative abundance of each mRNA of interest was assessed by real-time qRT-PCR using the SYBR Green Gene Expression Master Mix (Life Technologies, Carlsbad, CA, United States) in a StepOne Real-Time PCR System (Life Technologies, Carlsbad, CA, United States), as previously described (Caputi et al., 2020). Relative expression of different gene transcripts was calculated using the delta–delta Ct (ΔΔCt) method and converted to the relative expression ratio (2^−ΔΔCT^) for statistical analysis. All data were normalized to the housekeeping gene glyceraldehyde-3-phosphate dehydrogenase (*GAPDH*). The primers used for PCR amplification were designed using Primer3 and are reported in Table 1.

**TABLE 1:** Primer sequences for the genes tested by quantitative real-time polymerase chain reaction.

	Forward (5’-3’)	Reverse (3’-5’)
*PPAR-α*	TGGAGTCCACGCATGTGAAG	TTGTCGTACGCCAGCTTTAGC
*PPAR-γ*	CTGTTCGTACGCCAGCTTTAGC	GCTCATATCTGTCTCCGTCTTCTT
*KDM6A*	TTTGGTCTACTTCCATTACAATGCA	AAGCCCAAGTCGTAAATGAATTTC
*SOD-1*	CGACGAAGGCCGTGTGCGTGCTGAA	TGGACCACCAGTGTGCGCCCAATGA
*GAPDH*	AACTTTGGCATTGTGGAAGG	ACACATTGGGGGTAGGAACA

### 2.9 SDS-page and immunoblotting

PFC protein extraction was performed by the use of the T-PER Tissue Protein Extraction Reagent (Thermo Fisher Scientific, Waltham, MA, United States), following the manufacturer’s procedures. The Halt Protease and Phosphate Inhibitor Cocktail (Thermo Scientific) was added in accordance with the data sheet recommendations. Protein quantification was performed by the use of Pierce BCA Protein Assay Kit (Invitrogen Thermo Scientific). Proteins (50 μg) were separated in one dimension on Bolt 4%–12% Bis-tris Plus gels (Invitrogen Thermo Scientific) using a mini protean vertical gel electrophoresis mini-tank module (Invitrogen Thermo Scientific). Electrophoresis was performed for 3 h at 150 V at room temperature using the MOPS (1M Tris, 2% SDS, and 20 mM EDTA; pH 7.7) or MES (1M Tris, 20 mM EDTA, and 2% SDS; pH 7.3) purchased from Life Technologies, Thermo Fisher Scientific. A mixture of prestained protein standards (SeeBlue Plus-2 by Life Technologies, Thermo Scientific), with molecular weights ranging from 198 to 3 kDa, was loaded as the molecular weight marker. Proteins were transferred onto a 0.2-μm nitrocellulose membrane (Novex-Life Technologies, Thermo Fisher Scientific) at 10 V for 2 h using 25 mM Tris, 190 mM glycine, and 20% methanol as a transfer buffer. Non-specific binding sites on the membrane were blocked with Pierce Clear Milk Blocking buffer (Thermo Fisher Scientific). Primary antibodies were diluted in TBST skimmed milk 5% buffer, and the incubation was performed overnight at 4°C using an orbital shaker followed by 2 h at room temperature with the secondary horseradish peroxidase (HRP)-linked antibody (Goat Anti-Mouse or Anti-Rabbit IgG Peroxidase Conjugated by Thermo Fisher Scientific). Proteins that bound to the antibody were visualized by chemiluminescence procedures (Clarity Western ECL substrate Bio-Rad). The signal intensity of each lane was normalized to the α-tubulin loading control. The primary antibody working dilutions were determined following the manufacturer’s recommendations: mouse monoclonal antibody for α-tubulin (1:1,000), rabbit NRF2 (1:500) (ABclonal); clonal antibody purchased from Thermo Fisher Scientific; rabbit polyclonal antibody to IL-6 (1:500) (ABclonal); rabbit polyclonal antibody to IL-8 (1:500) (ABclonal); rabbit polyclonal antibody to NF-κB (phospho-Ser 536) (1:500) (Bioss); rabbit polyclonal antibody to Nf-kB (1:500) (Bioss); rabbit polyclonal antibody to (phospho-Ser 536) (1:500); rabbit polyclonal antibody to NF-κB (1:500); rabbit polyclonal antibody to TNF-α (1:500) (ABclonal); rabbit polyclonal antibody to IL-1β (1:500) (ABclonal); rabbit polyclonal antibody to H2AX (phospho Ser 139) (Cusabio) (1:500); rabbit polyclonal antibody to H2AX (1:500) (Cusabio); rabbit polyclonal antibody to OGG-1 (1:500) (Elabscience); rabbit polyclonal antibody to xeroderma pigmentosum group C protein (XPC) complex (Cloud-Clone Corp.) (1:500); rabbit polyclonal antibody to CYP 1A1, 2A6, 2B6, and 2E1 (1:500) (Cloud-Clone Corp.); rabbit polyclonal antibody to SOD-1 (1:500) (Cloud-Clone Corp.); and rabbit polyclonal antibody to CAT (1:500) (Cloud-Clone Corp.). Gels were run in duplicate, and the results represented the average from two different runs. Original immunoblots are presented in the Supplementary Material.

### 2.10 Statistical analysis

Data were evaluated using the Shapiro–Wilk tests to confirm the normality of the distribution and using Grubb’s test to identify outliers. Statistical analysis was performed using the two-tailed unpaired *t*-test or Mann–Whitney test in case of non-normality distribution. All statistical analyses were performed using GraphPad 9 software (San Diego, CA, United States). The results are expressed as the means ± standard error mean (SEM) (*n* = 4–6 animals/group). *p*-values < 0.05 were considered statistically significant: ^*^ *p < 0.05*; ^**^ *p < 0.01*; ^***^ *p < 0.001*; ^****^ *p < 0.0001*.

## 3 Results

### 3.1 Chemical characterization of the HnB mainstream

**FIGURE 1:**
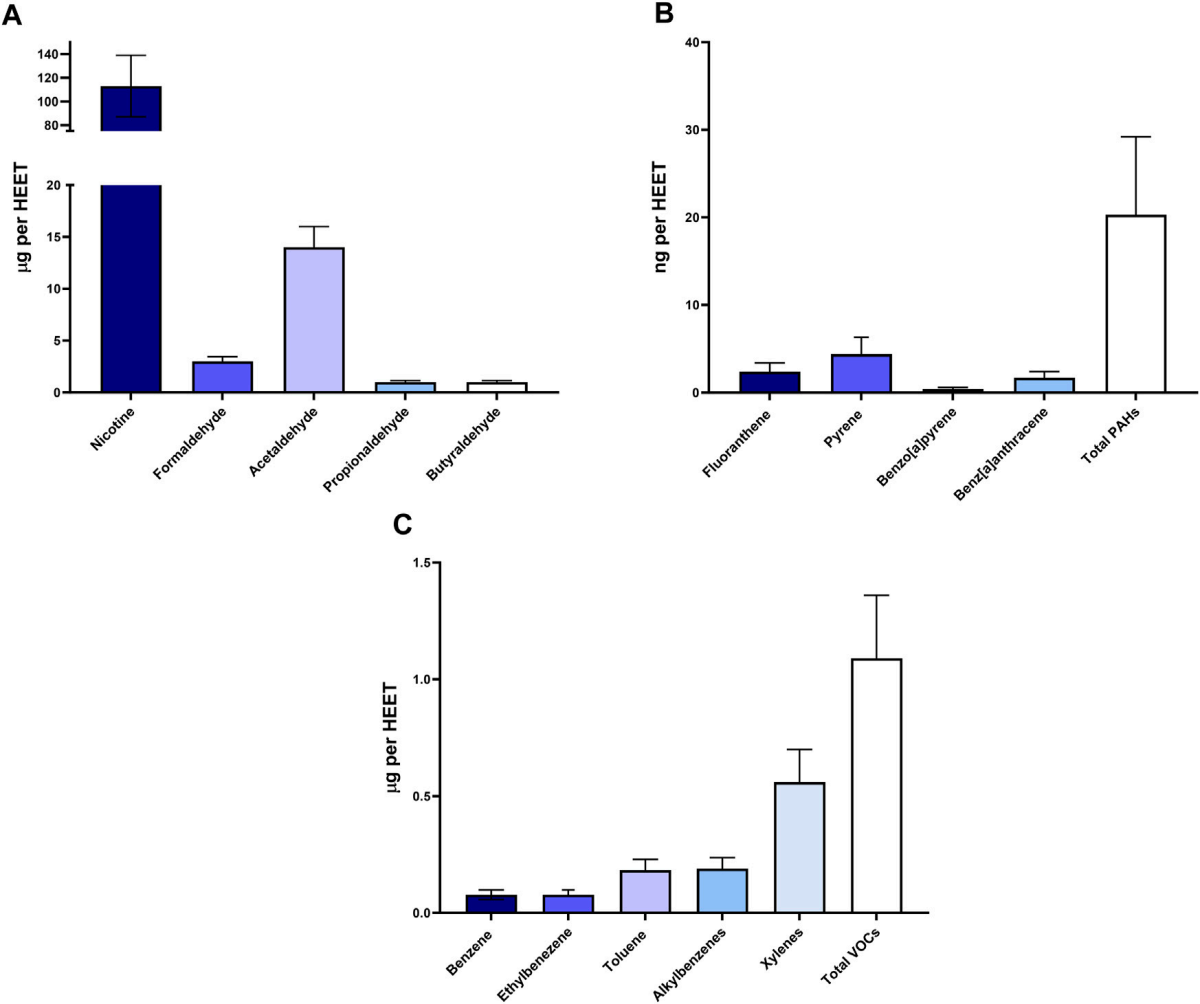
Chemical analysis of HnB smoke. Data show the presence of toxic aldehydes **(A)**, polycyclic aromatic hydrocarbons **(B)**, and volatile organic compounds **(C)**. Data are expressed as the means ± SD of at least two replicates from two independent experiments.

We characterized HnB mainstream using GC-MS based on representative tobacco smoke carcinogens (Figures 1A–C), which revealed the presence of aldehydes and polycyclic aromatic hydrocarbons. These compounds are indicative of thermal degradation and incomplete combustion of tobacco. The concentration of nicotine recorded in the mainstream was significantly lower than the LC_50_ for vaporized nicotine in the rat model (2.3 mg/L).

### 3.2 Reactive radical species (RRS) production and gene/protein levels of antioxidant mediators

**FIGURE 2:**
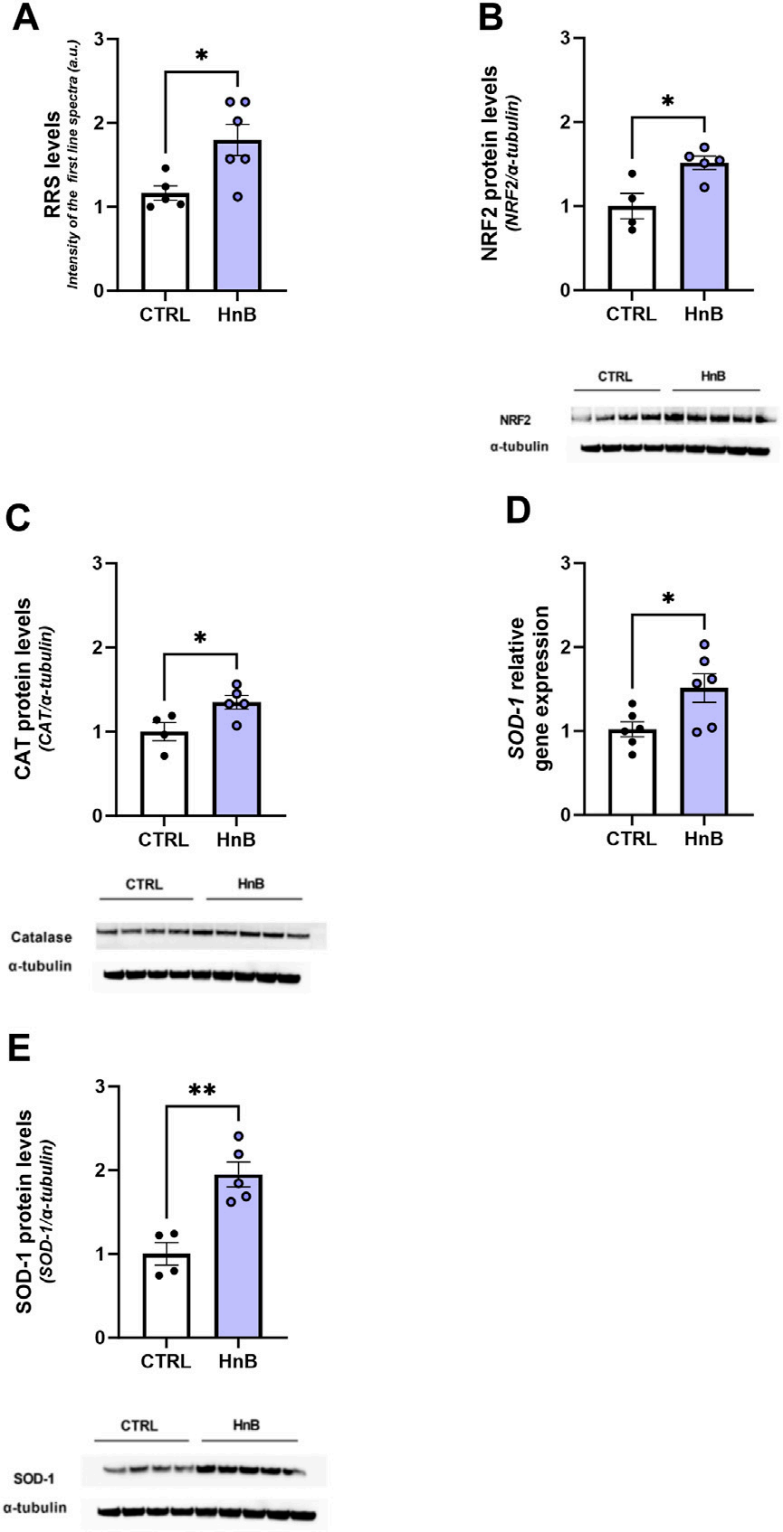
HnB exposure leads to oxidative stress and induces the NRF2-mediated antioxidant response. Radical species content, here measured through the intensity of the first spectral line of electronic paramagnetic resonance (EPR) (arbitrary units), is significantly higher in samples from exposed animals compared to those from controls **(A)**. HnB group showed a significant increase in NRF2 (∼65 kDa) **(B)**, with an upregulation of CAT (∼55 kDa) **(C)** and SOD-1, here shown as a relative gene expression **(D)** and protein expression (∼19 kDa) **(E)**. A representative blot is reported under the histograms. Bars represent the means ± SEM; ^*^ *p* < 0.05, ^**^ *p* < 0.01; two-tailed *t*-test.

A significant increase in the RRS content was observed in the PFC of rats exposed to HnB aerosol (1.80 ± 0.18 vs 1.16 ± 0.08, *p* < 0.01; Figure 2A).

Increased levels of the transcription factor NRF2 (1.51 ± 0.08 vs 1.00 ± 0.15, *p* < 0.05; Figure 2B) along with the antioxidant enzyme CAT (1.35 ± 0.08 vs 1.00 ± 0.11, *p* < 0.05; Figure 2C) and SOD-1 (1.95 ± 0.15 vs 1.00 ± 0.13, *p* < 0.01; Figure 2E) protein levels was also detected in the exposed group.

Moreover, an upregulation of SOD-1 mRNA levels was also observed (1.51 ± 0.17 vs 1.02 ± 0.09, *p* < 0.05; Figure 2D).

### 3.3 DNA oxidative damage and protein levels of DNA repair enzymes

**FIGURE 3:**
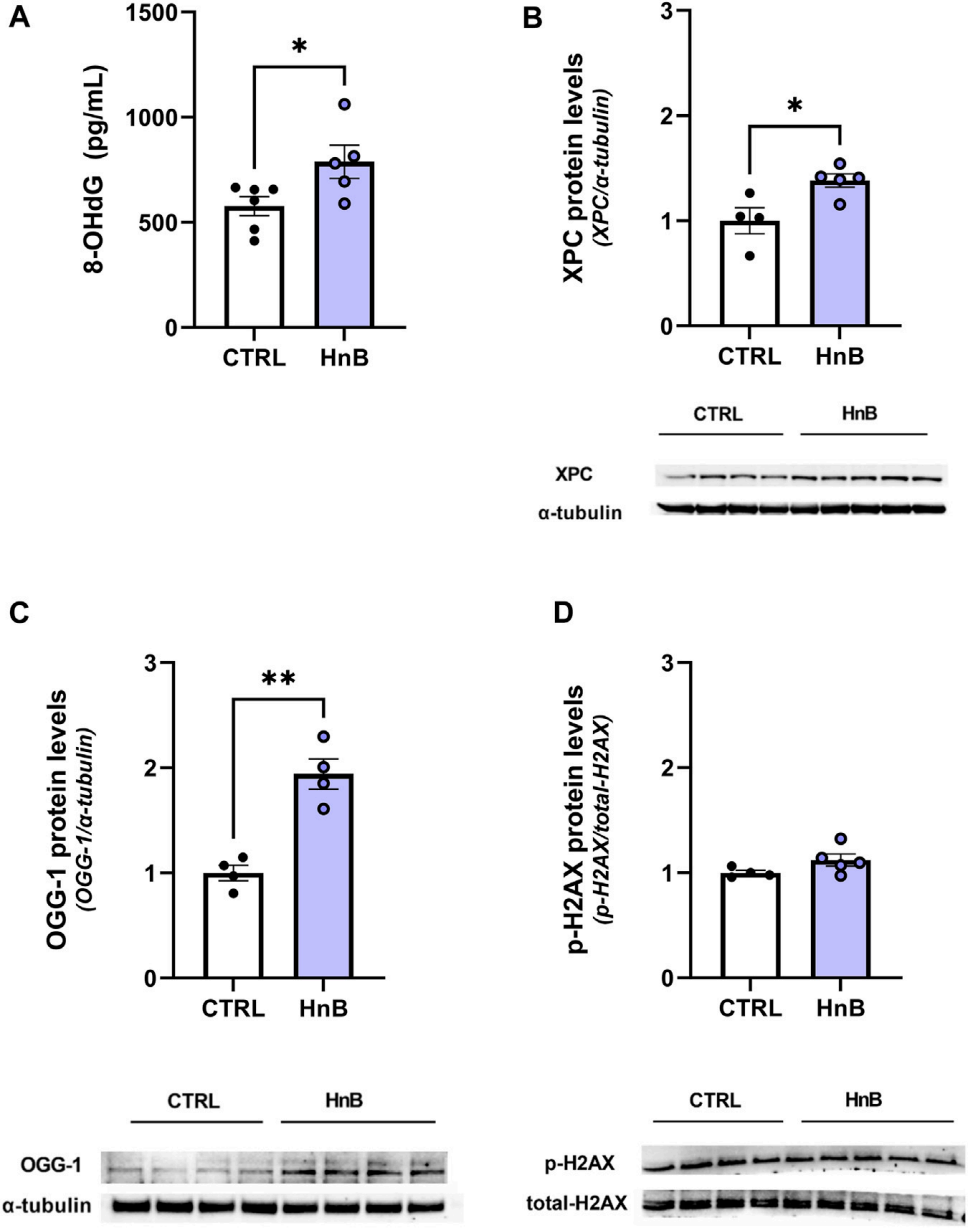
Animals exposed to HnB mainstream smoke show higher levels of 8-OHdG, a DNA oxidative damage marker, along with the activation of the DNA repair machinery through the induction of XPC and OGG-1 proteins. Exposed animals show higher levels of 8-OHdG, an oxidative damage marker, which is measured through ELISA assay and expressed as pg of 8-dG/mLOH **(A)**. DNA repair systems are induced in the exposed group: XPC (∼26 kDa) **(B)** and OGG-1 (∼47 kDa) **(C)** play a key role in nucleotide excision repair and base excision repair. They are significantly upregulated in the PFC of the exposed rats compared to controls. No significant changes in the H2AX (∼17 kDa) phosphorylation rate were recorded **(D)**. A representative blot is reported under the histograms. Bars represent the means ± SEM; ^*^ *p* < 0.05, ^**^ *p* < 0.01; two-tailed *t*-test.

Student’s t-test showed significantly higher levels of the oxidative damage marker, 8-OHdG (788.03 ± 78.70 vs. 576.69 ± 44.94, *p* < 0.05; Figure 3A), in the PFC of rats exposed to HnB technology.

Consistently, an increase in the expression levels of the DNA repair enzymes xeroderma pigmentosum group C protein complex (1.38 ± 0.06 vs 1.00 ± 0.13, *p* < 0.05; Figure 3B) and 8-oxoguanine DNA glycosylase-1 (OGG-1) (1.94 ± 0.27 vs 1.00 ± 0.19, *p* < 0.01; Figure 3C) was also detected. No significant changes in the phosphorylation of histone H2AX at the Ser139 residue were been observed (1.12 ± 0.06 vs 1.00 ± 0.02, *p* > 0.05; Figure 3D) in the animals of the smoking group compared to those in the control group.

### 3.4 Protein alterations of cytochrome P450 (CYP) isoforms

**FIGURE 4:**
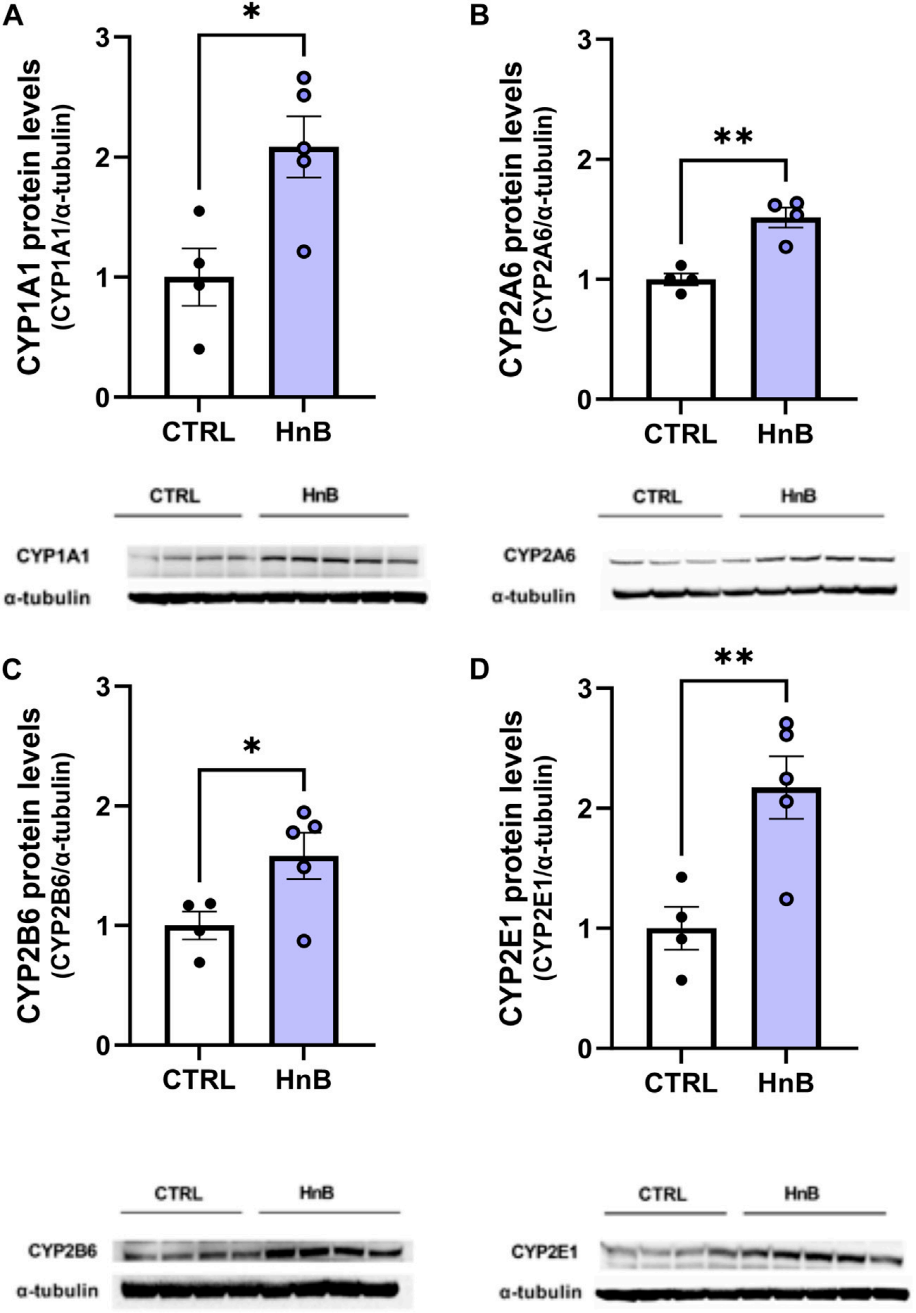
HnB exposure induces the expression of CYPs. The exposure to HnB smoke led to an overexpression of CYP1A1 (∼63 kDa) activating aromatic amines, dioxins, and PAHs **(A)**. CYP2A6 (∼56 kDa) (involved in metabolic activation of carcinogenic nitrosamines in tobacco smoke as well as nicotine metabolism); **(B)**; CYP2B6 (∼66 kDa) (activating bupropion smoking-cessation drug); **(C)** and CYP2E1 (∼50 kDa) (activating alcohol, nitrosamines, benzene, acetone, and acrylamide) **(D)**. A representative blot is reported under the histograms. Bars represent the means ± SEM; ^*^ *p* < 0.05, ^**^ *p* < 0.01; two-tailed *t*-test.

A significant increase (*p* < 0.05) in CYP1A1 isoform protein levels in the PFC of rats exposed to tobacco aerosol delivered by HnB technology was observed in respect to the control group animals (2.09 ± 0.25 vs 1.00 ± 0.24, *p* < 0.05; Figure 4A).

Analogously, increased levels of CYP2A6 (1.52 ± 0.08 vs 1.00 ± 0.05, *p* < 0.01; Figure 4B), CYP2B6 (1.58 ± 0.19 vs 1.00 ± 0.11, *p* < 0.05; Figure 4C), and CYP2E1 (2.17 ± 0.26 vs 1.00 ± 0.18, *p* < 0.01; Figure 4D) protein levels were detected in the exposed animals compared to control animals.

### 3.5 Protein alterations of the protooncogene c-MYC

**FIGURE 5:**
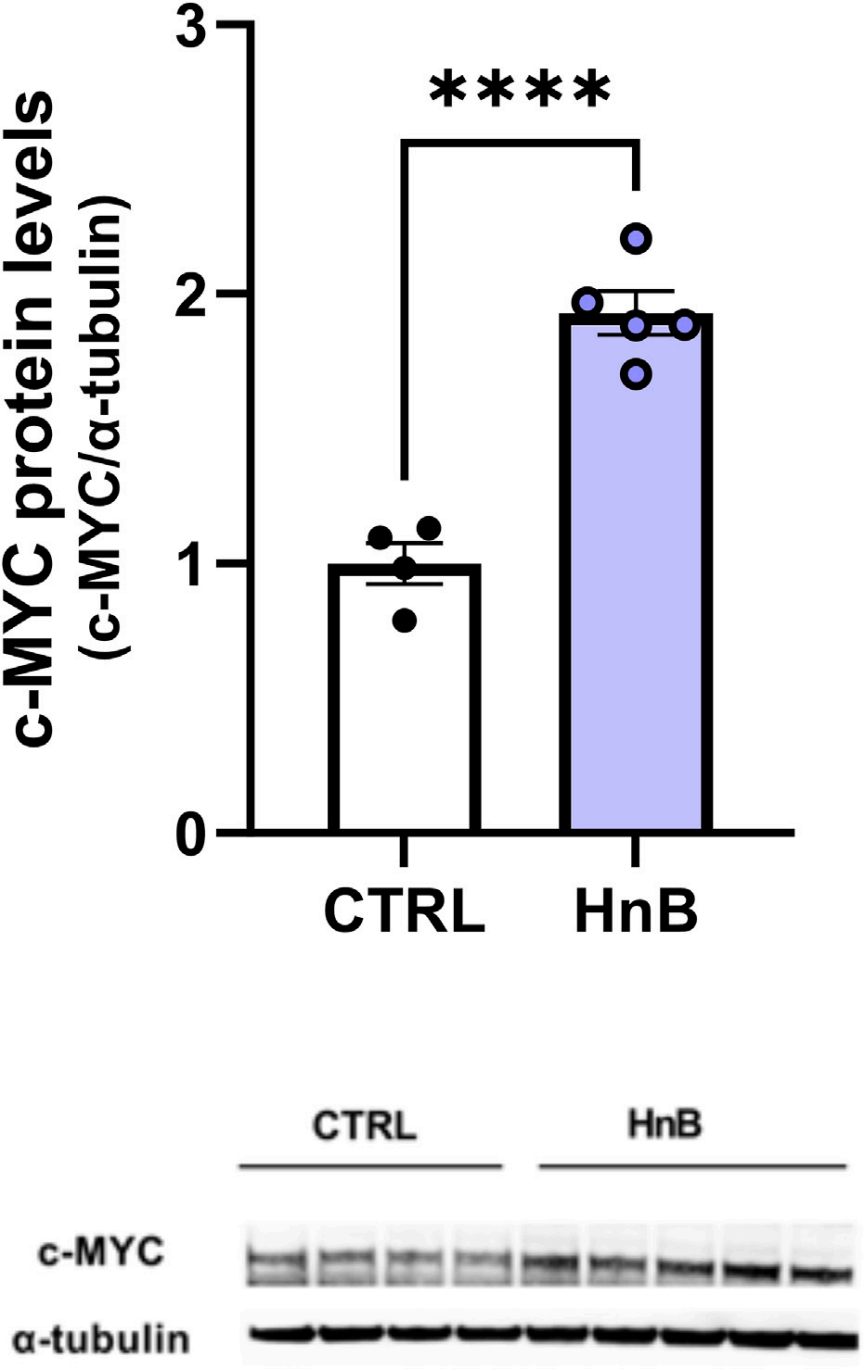
HnB exposure induces the expression of protooncogene c-MYC. Animals exposed to HnB smoke showed an increase in protooncogene c-MYC (∼49 kDa). A representative blot is reported under the histograms. Bars represent the means ± SEM; ^****^ *p* < 0.0001; two-tailed *t*-test.

The unpaired *t*-test revealed a significant increase in the protooncogene c-MYC protein levels in the PFC of rats exposed to tobacco aerosol delivered by HnB technology in respect to the animals in the control group (1.92 ± 0.08 vs 1.00 ± 0.08, *p* < 0.0001; Figure 5).

### 3.6 Protein alterations of inflammatory mediators

**FIGURE 6:**
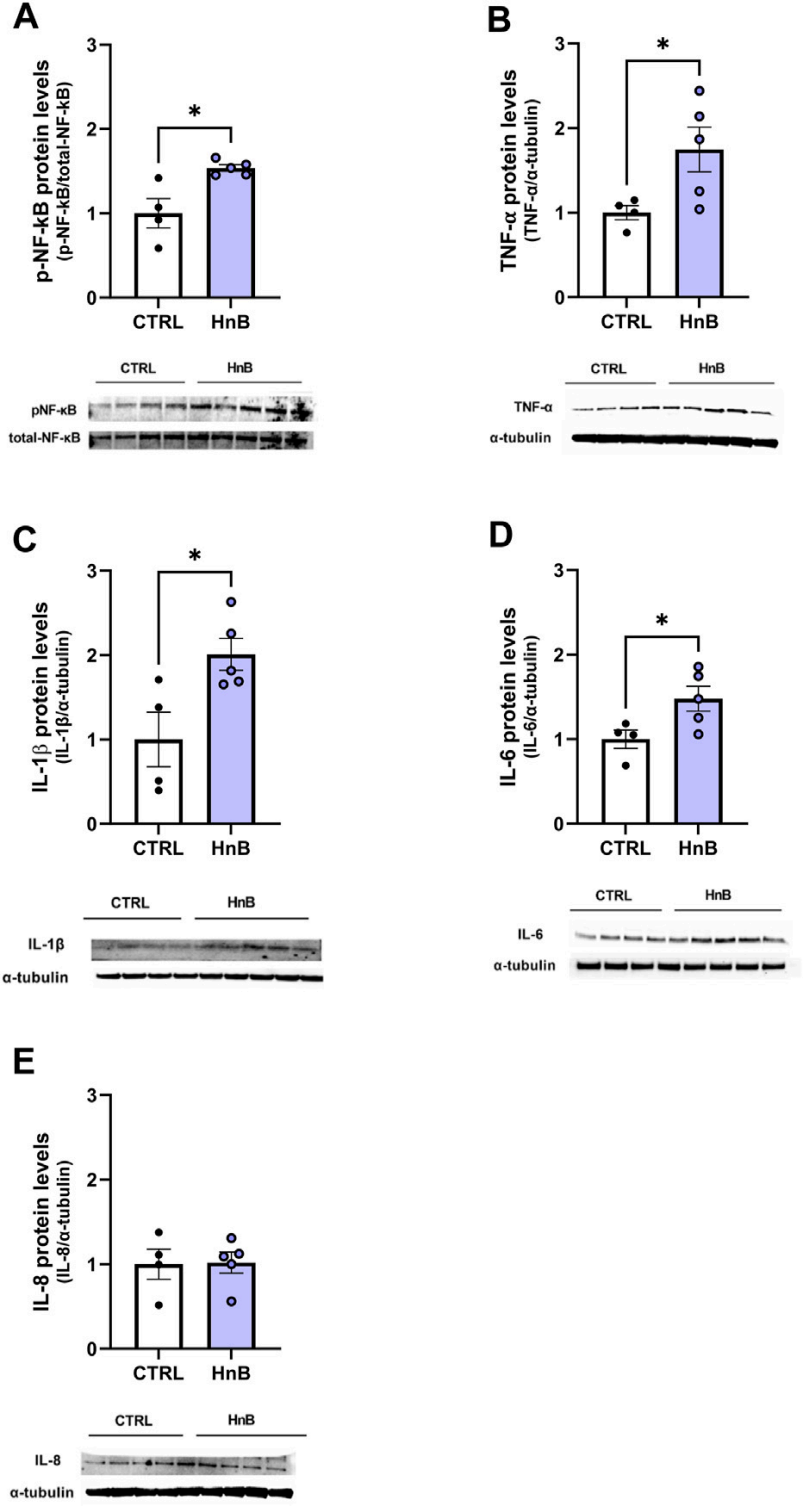
HnB activates NF-κB-mediated inflammatory response. Exposed animals reported an increase in phosphorylation of NF-κB (p65 Ser 536) (∼61 kDa) **(A)**, along with the upregulation of TNF-α (∼25 kDa) **(B)**, IL-1β (∼17 kDa) **(C)**, and IL-6 (∼26 kDa) **(D)**. On the contrary, no significant changes in IL-8 (∼11 kDa) **(E)** expression were observed. A representative blot is reported under the histograms. Bars represent the means ± SEM; ^*^ *p* < 0.05; two-tailed *t*-test.

Significantly higher levels of phosphorylated NF-κB protein levels (1.53 ± 0.04 vs 1.00 ± 0.17, *p* < 0.05; Figure 6A) were observed in the PFC of rats exposed to HnB technology compared to the control group. Moreover, data analysis revealed a significant increase in the expression of the pro-inflammatory mediators TNF-α (1.75 ± 0.26 vs 1.00 ± 0.08, *p* < 0.05; Figure 6B), IL-1β (2.01 ± 0.19 vs 1.00 ± 0.32, *p* < 0.05; Figure 6C), and IL-6 (1.48 ± 0.15 vs 1.00 ± 0.11, *p* < 0.05; Figure 6D) in the animals of the smoking group compared to those in the control group. No significant changes in the protein levels of IL-8 (1.02 ± 0.12 vs 1.00 ± 0.18, *p* > 0.05; Figure 6E) were observed.

### 3.7 Gene expression alterations of nuclear receptors PPARs and epigenetic enzyme KDM6A

**FIGURE 7:**
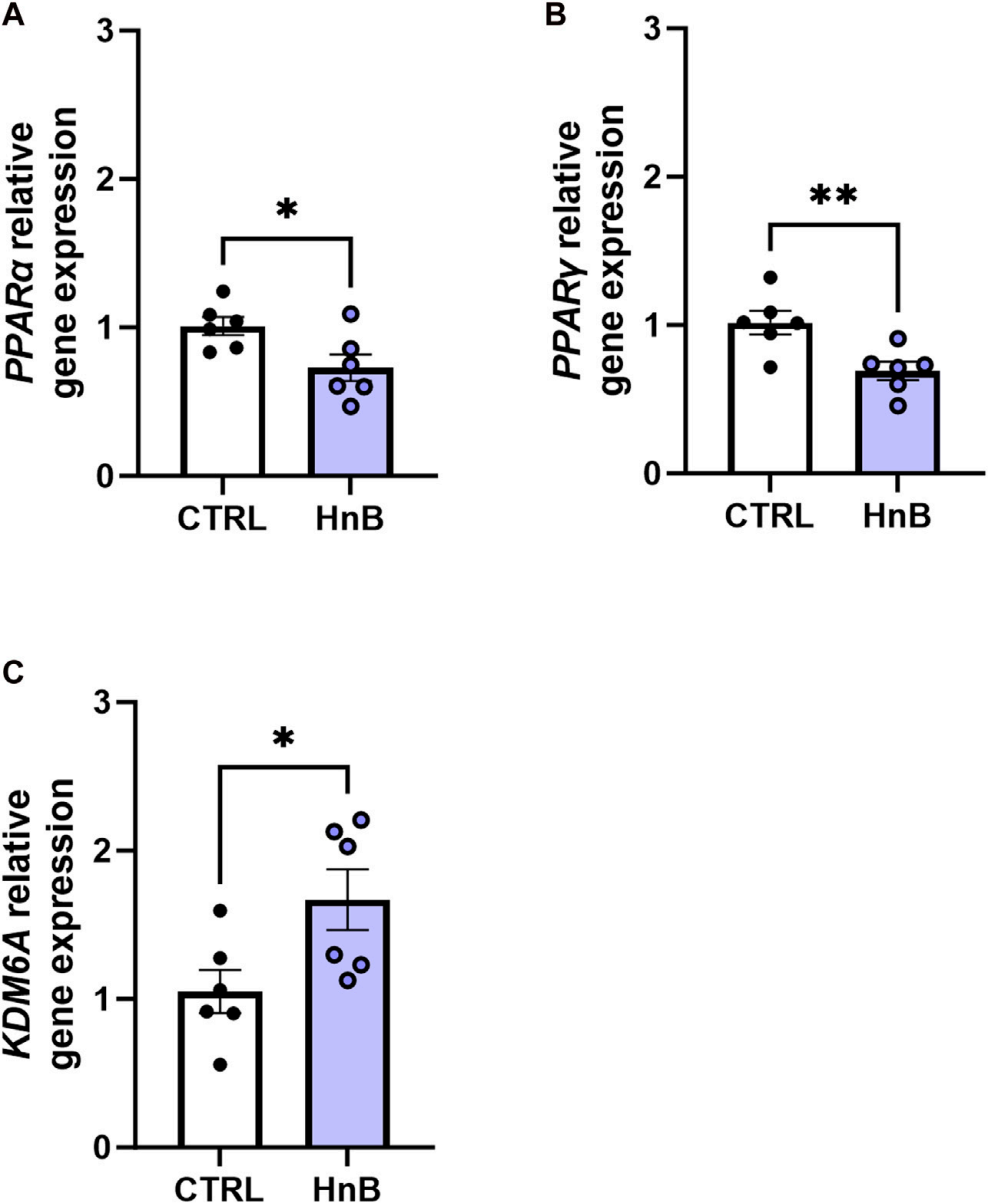
Effects of the HnB mainstream on the relative gene expression of KDM6A **(A)**, PPARα **(B)**, and PPARγ **(C)** in the rat PFC. Data represent 2^−ΔΔCT^ values calculated using the ΔΔCT method and are expressed as the means ± SEM; ^*^ *p* < 0.05; ^**^ *p* < 0.01, two-tailed *t*-test.

A downregulation in mRNA levels of the nuclear receptors PPARα (0.73 ± 0.09 vs 1.01 ± 0.06, *p* < 0.05; Figure 7A) and PPARγ in the PFC of rats in the smoking group (0.69 ± 0.06 vs 1.02 ± 0.08, *p* < 0.01; Figure 7B) was observed.

Interestingly, increased gene expression levels of the epigenetic enzyme histone demethylase, KDM6A (1.67 ± 0.20 vs 1.05 ± 0.14, *p* < 0.05; Figure 7C), were detected in rats exposed to tobacco aerosol delivered by HnB technology compared to those in the control group.

## 4 Discussion

Aggressively advertised as almost risk-free aids for smokers, the new electronic tobacco products to which heat-not-burn devices belong have an impressive appeal among youth and young adults due to the industry claims that they are safer than traditional tobacco cigarettes while maintaining the typical gesture of the latter and nicotine intake. Since it takes decades for exposure to smoke to induce chronic diseases in humans, including cancer, animal models are imperative to address the question of whether HnB might pose a public health risk. Some emerging findings seem to indicate that e-cigarette consumption could lead to transcriptome alterations associated with adverse neurobiological outcomes along with loss of blood–brain barrier (BBB) integrity and changes in brain lipidome (Cardenia et al., 2018; Ruszkiewicz et al., 2020); however, data on the potential neurotoxic effects of HnB devices are still scarce. Due to thermal degradation or incomplete combustion of tobacco stick, HnB mainstream smoke contains many chemical compounds such as aldehydes and polycyclic aromatic hydrocarbons, which are widely recognized as neurotoxic and carcinogenic (Vivarelli et al., 2021). Furthermore, according to recent studies, the presence of tobacco pyrolysis or thermodegradation markers such as formaldehyde, acetaldehyde, volatile organic compounds, and PAHs (e.g., benzo [a]pyrene) questions the affirmations of the “smoke-free” mainstream (Uguna and Snape, 2022).

Here, we show that HnB exposure significantly increases RRS production in the prefrontal cortex of adult rats. This result, consistent with a previous study using both traditional and electronic cigarettes, highlights that HnB devices could promote the oxidative stress (Sivandzade and Cucullo, 2019). This observation is also supported by increased protein levels of the transcription factor NRF2 and the antioxidant enzymes catalase (CAT) and superoxide dismutase (SOD-1); for this latter enzyme, mRNA levels were also boosted. In this frame, these last changes could suggest the activation of an adaptive mechanism aimed at counteracting the excessive radical production.

Our data also show that the exposure to HnB induced a significant NF-κB phosphorylation in the PFC. Consistently, higher levels of TNF-α, IL-1β, and IL-6 proteins were detected in the PFC area of treated rats compared to control animals. Together with the increase in RRS, the present results strongly indicate that HnB could trigger inflammation in specific brain areas other than what has already been reported for peripheral tissues such as the lungs and upper airways (Vivarelli et al., 2021). Although some evidence about nicotine stimulation of IL-8 expression has been reported *in vitro* (Iho et al., 2003), our results did not show any significant alterations in IL-8 protein levels, thus suggesting a minor impact of HnB on IL-8 in the PFC.

An upregulation of the histone demethylase KDM6A gene expression was also detected in our experimental condition in the investigated brain area. This result is consistent with the relevant role proposed for demethylation enzymes in ensuring inflammatory response. Indeed, the KDM6A-mediated removal of the repressive tri-methylation mark (H3K27me3) in lysine-27 of histone H3 is crucial for NF-κB-dependent inflammatory gene regulation (Higashijima et al., 2020). Moreover, the concomitant increase in IL-6/IL-1β protein levels is in agreement with the capacity of KDM6A to epigenetically drive the production of these cytokines (Sivandzade and Cucullo, 2019), which, in turn, participate in the dysregulation of interendothelial junctions and promote leukocyte adhesion and migration (Vardam et al., 2007). In this regard, loss of blood–brain barrier integrity has been highlighted as a major event involved in smoking-induced neurotoxicity, associated with both traditional tobacco smoking and e-cigarette use (Sivandzade and Cucullo, 2019).

Interestingly, HnB mainstream also caused significant downregulation of both PPARα and PPARγ nuclear receptors. Much evidence identified PPARs as negative regulators of oxidative stress-induced inflammation (Ding et al., 2020). Indeed, *in vivo* and *in vitro* studies have shown that PPARγ is able to reduce the production of downstream inflammatory factors by inhibiting the NF-κB pathway (Ding at., 2020). Moreover, the KDM6A ability to reduce PPAR expression and activity has been recently reported (Rullo et al., 2021).

**FIGURE 8:**
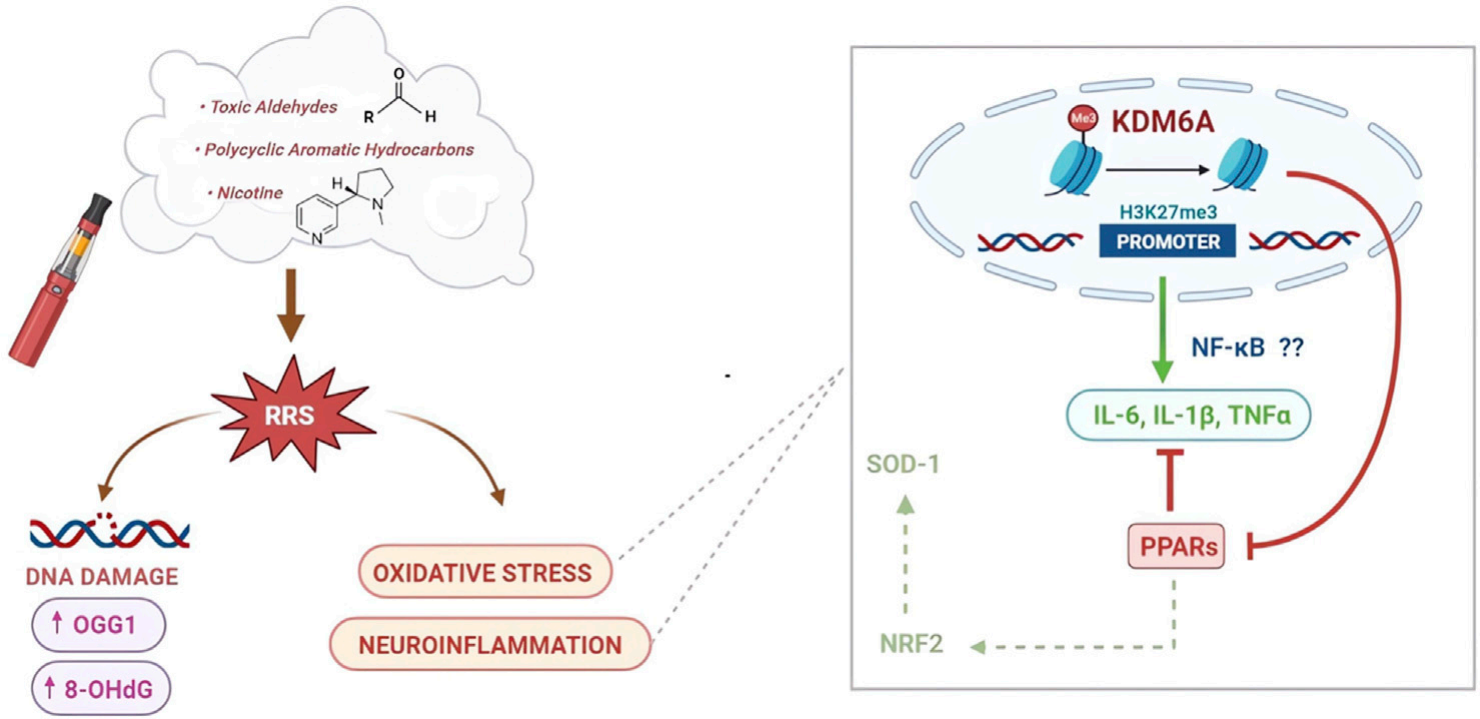
Schematic representation of the effects of HnB smoking on oxidative stress, inflammatory process, and DNA damage. A large number of compounds found in the HnB mainstream are oxidants or chemicals that can be bioactivated by CYP to mutagens and ultimate carcinogens (via carcinogenesis) (center). Many of them are known inducers of CYP (e.g., aldehydes and PAHs), a phenomenon typically associated with both a greater bioactivation of ubiquitous pre-mutagens/pre-carcinogens and an increase in the generation of ROS (via co-carcinogenesis) (left side). Both processes can increase the risk of cancer, the former directly due to the effect of smoke compounds and the latter indirectly through the upregulation determined by these same compounds. Moreover, we can assume that oxidative stress promoted neuroinflammatory processes through an upregulation of histone demethylase KDM6A by increasing the target NF-κB pro-inflammatory mediators, such as IL-6 and IL-1β, together with the downregulation of PPAR expression as negative regulators of oxidative stress-induced inflammation (right side).

These observations emphasize that HnB smoke can promote neuroinflammatory processes in the prefrontal cortex. This effect includes increased generation of ROS and dysregulation in the expression of transcription factors (NRF2 and NF-κB), cytokines (IL-6, IL-1β, and TNF-α), and precise nuclear receptors (PPARα and PPARγ), which are all mutually regulated elements clearly involved in the physiological maintenance of the cellular redox balance (Ding et al., 2020). Interestingly, the epigenetic regulation of some of these elements involves the action of histone demethylase enzymes such as KDM6A, whose mRNA levels have been found to be altered by HnB smoke (Figure 8).

These results also draw attention to the possible involvement of inflammatory processes in nicotine addiction dependence. Indeed, it has recently been argued that neuroinflammation participates in the development of drug addiction (Kohno et al., 2019; Johnstone et al., 2021). In particular, the role of KDMs in inflammatory signaling pathways that contribute to neurochemical changes in the reward circuitry activated by drugs of abuse has been proposed (Johnstone et al., 2021).

While nicotine cannot bind to DNA directly and its metabolite cotinine is generally considered nontoxic and noncarcinogenic (Lee et al., 2018), the presence of well-known powerful carcinogenic agents such as PAHs and aldehydes in HnB tobacco stick smoke along with the high observed RRS content in the PFC supports the hypothesis that exposure to this electronic cigarette promotes cancer risk. On the other hand, our model shows an increased level of 8-hydroxyguanosine, a reliable marker of DNA oxidation that is positively associated with smoking habit (Graille et al., 2020). Consistently, the xeroderma pigmentosum group C protein complex and 8-oxoguanine DNA glycosylase-1 (OGG-1), two crucial proteins involved in nucleotide excision repair (NER) and DNA base excision repair (BER), respectively (Tang et al., 2022), are upregulated in the exposed group. Interestingly, our data show no significant changes in the phosphorylation of histone H2AX at the Ser139 residue (γ-H2AX), early cellular responses of DNA double-strand breaks which is considered a biomarker for the toxicological risk assessment of tobacco products (Sakurai et al., 2022). However, it was recently demonstrated that aldehydes such as formaldehyde can inhibit the activation of H2AX phosphorylation pathways (Yang et al., 2019), and the chemical characterization of HnB mainstream smoke here reports that aldehydes are the chemical species with the highest concentration after nicotine. It is therefore plausible that the aldehydes blocked the activation of H2AX. These observations are consistent with recent evidence showing that HnB exposure resulted in no changes in H2AX activation *in vitro* (Rahman et al., 2022), while OGG-1 increased significantly.

Because tobacco smoke increases brain expression of carcinogenic bioactivating cytochrome P450 (CYP) isoforms 1A1 (activating polychlorinated biphenyls, aromatic amines, PAHs, and alkylnitrosamines), 2E1 (activating alcohol, nitrosamines, benzene, acetone, and acrylamide), and 2B6 and 2A6 in both animal models and humans (Zevin and Benowitz, 1999), we hypothesized that similar changes would occur with HnB smoking. In the PFC of HnB tobacco-exposed animals, we found a marked increase in the expression of the isoforms CYP1A1, CYP2A6, CYP2B6, and CYP2E1, suggesting an increase in the pre-carcinogen biotransforming potential of the users (Paolini et al., 1999).

The CYP2 family metabolizes a large proportion of CNS-acting drugs, such as bupropion, diazepam, methadone, sertraline, and tramadol, and some endogenous neurochemicals, such as dopamine and serotonin, and brain CYPs are often regulated very differently from the hepatic forms; brain CYP2B6 is elevated in smokers, while liver CYP2B6 is unaffected by smoking. Extrapolated to humans, these data suggest that HnB consumers may respond differently from never-smokers to CNS-acting drugs or neurotoxins that are CYP2B6 substrates, without difference in plasma levels, similar to those observed in smokers who usually require a higher dose of propofol to achieve anesthesia (Miksys and Tyndale, 2013). Again, changes in brain CYP2B6 expression may lead to increased metabolism and impaired duration of action of bupropion, thereby affecting outcomes of smoking cessation treatment (Lee et al., 2007). Upregulation of CYP2B6 can also increase the toxicological risk associated with the environmental exposure of some pesticides such as chlorpyrifos, which is converted to the neurotoxic oxon metabolite mainly by CYP2B6 (Herriage et al., 2022). Interestingly, increased CYP2E1 expression was observed in the PFC of alcoholics and smokers and was associated with ethanol and nicotine dependence (García-Suástegui et al., 2017); in addition, evidence from animal models indicates that CYP2E1 induction exacerbates neurological deficit and increases oxidative stress, inflammation, and neurodegeneration (Yu et al., 2021). On the other hand, CYP induction, regardless of the induced isoform, contributes to the formation of ROS (co-carcinogenesis), due to the uncoupling of the CYP–Fe II–O_2_ complex of the CYP catalytic cycle (Paolini et al., 1996). In the light of these findings and considering the critical role of PPARs in nicotine dependence (Domi et al., 2019), it is possible to hypothesize that the increase in CYP2E1 could participate, via PPAR downregulation, in the development of neuroinflammatory processes and remodeling of synaptic plasticity that are also likely involved in tobacco addiction (Na et al., 2017; Butler et al., 2021). The increased levels of CYP2A6 further support this hypothesis. Indeed, some evidence suggests a positive correlation between CYP2A6 and nicotine reinforcement, which results in increased smoking behaviors and decreased cessation efforts (Butler et al., 2021). Furthermore, increased expression levels of CYP1A1 (activating polychlorinated biphenyls, aromatic amines, PAHs, and alkylnitrosamines) could pose a tipping issue as it is positively associated with various malignancies including brain cancer. In this regard, smoking is associated with an increased risk of glioma (Ahn et al., 2020), and several studies exploring the molecular mechanisms involved in cigarette smoke-mediated cell proliferation have highlighted the upregulation of the c-MYC protein (Schaal and Chellappan, 2014). On these bases, we therefore considered the hypothesis of a putative change in c-MYC regulation, and the results of our model showed a significant increase in c-MYC expression in the exposed group compared to controls.

The protooncogene c-MYC is overexpressed in the most malignant primary brain tumor, glioblastoma multiforme (GBM), and is considered essential for GB transformation by either increasing the sensitivity of astrocytes to gliomagenesis (Lassman et al., 2004) or influencing the brain tumor microenvironment by boosting the expression of inflammatory mediators such as IL-1β (Shchors et al., 2006). Pivotal studies suggest that MYC inhibition could prevent glioma formation, blocking cell proliferation and survival and even inducing disease regression.

In conclusion, our findings suggest that, although the HnB device releases a concentration of carcinogenic and neurotoxic compounds lower than those usually present in cigarette smoke, HnB consumption mimics some pathological mechanisms typically triggered by conventional cigarettes, including oxidative DNA damage, co-carcinogenesis, and the deregulation of cellular pathways associated with neuroinflammation and neurodegeneration. Moreover, the alterations reported here suggest that nicotine, similar to other drugs of abuse (e.g., cocaine and ethanol), may be able to alter oxidative and neuroinflammatory mediators, which are known to play a key role in the molecular mechanism associated with the development of substance use disorders (SUDs). In this regard, recent evidence from clinical surveys seems to confirm the hypothesis that HnB consumption may result in increased tobacco consumption or dual-use with cigarettes (Stone et al., 2022).

Although the study lacks a comparison between HnB and traditional tobacco cigarette and further investigation will be useful to address the potential toxicity of these novel nicotine delivery systems, the present study suggests that caution is warranted when HnB is proposed as a healthy alternative to smoking and/or an aid to smoking cessation.

## Data Availability

The original contributions presented in the study are included in the article/Supplementary Material; further inquiries can be directed to the corresponding author.
