# Supplementary material for: Effects of unburned tobacco smoke on inflammatory and oxidative mediators in the rat prefrontal cortex
Source: Front Pharmacol. 2024 Jan 25;15:1328917. doi: 10.3389/fphar.2024.1328917 (PMC10851081; doi:10.3389/fphar.2024.1328917)
Supplement: Supplementary file 1 [file Table1.DOCX]

Supplementary Material

## Western blot images

Lines 1-4: CTRL; Lines 5-9: HnB

Lines 1-4: CTRL; Lines 5-9: HnB


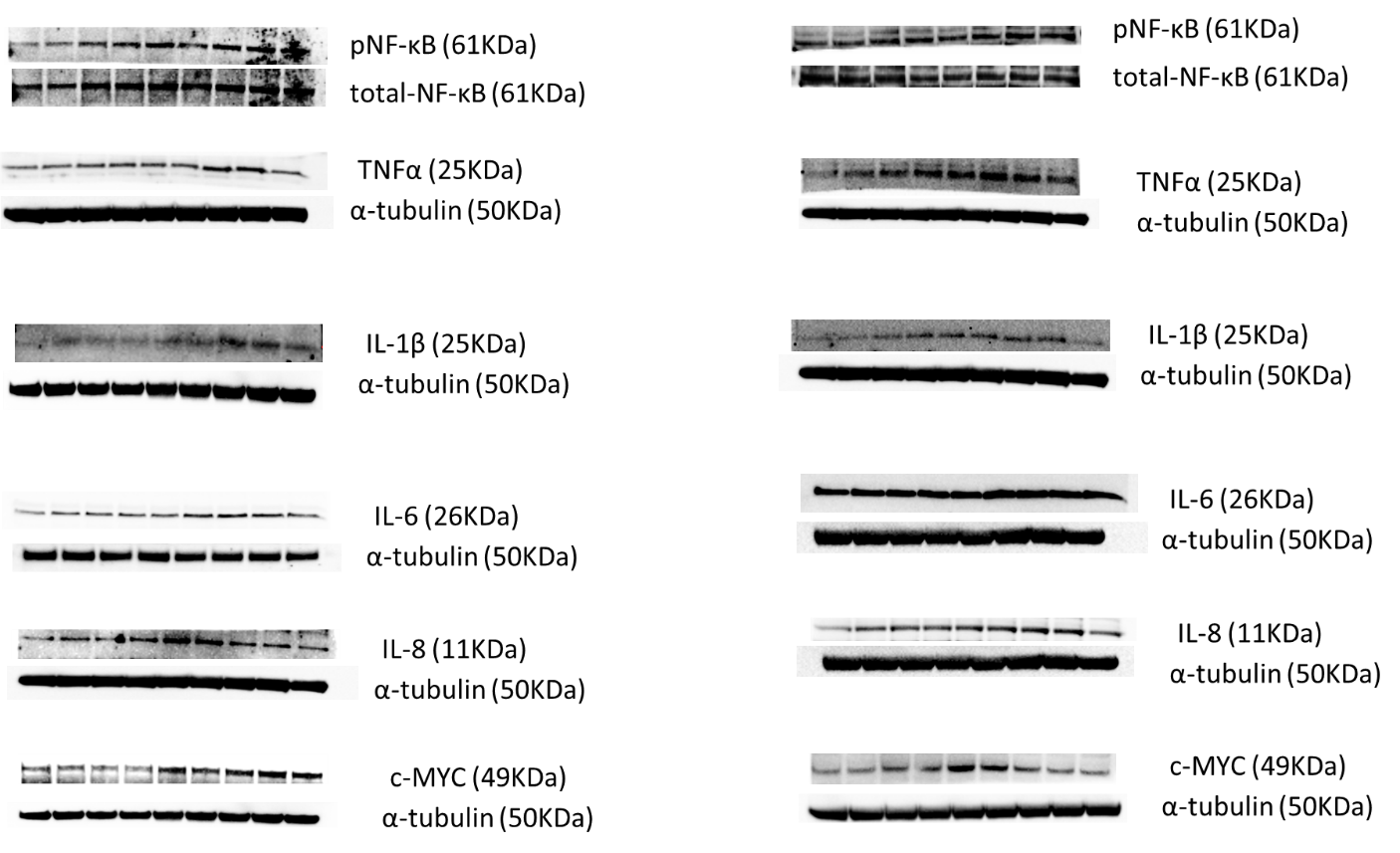


Lines 1-4: CTRL; Lines 5-9: HnB
